# Effect of surface treatments with acid solutions on the surface roughness of an Yttrium-Tetragonal Zirconia Polycrystal

**DOI:** 10.4317/jced.54571

**Published:** 2018-04-01

**Authors:** Frederico Goyatá, Yvna Galvão, Thamyryz-Rafaela Simões, Luiz-Felipe Goyatá, José-Alcides Arruda, Amália Moreno

**Affiliations:** 1 Department of Oral Surgery, Pathology and Clinical Dentistry; School of Dentistry, Universidade Federal de Minas Gerais (UFMG), Belo Horizonte, MG, Brazil; 2 Department of Restorative Dentistry, São Leopoldo Mandic Institute and Dental Research Center, Campinas, SP, Brazil; 3 Department of Restorative Dentistry, São Leopoldo Mandic Institute and Dental Research Center, Campinas, São Paulo, SP, Brazil

## Abstract

**Background:**

The purpose of this *in vitro* study was to evaluate the effect of conventional surface treatment with acid solutions on the surface roughness of a zirconia-based ceramic.

**Material and Methods:**

Specimens of yttrium-tetragonal zirconia polycrystal (Y-TZP) -based ceramic were fabricated (5.0 x 5.0 x 2.0 mm, n=40). The specimens were submitted to the tested surface treatment method and divided into 4 groups (n=10): no treatment-control (GI), airborne 110 µm aluminum oxide particle abrasion for 1 minute-conventional method (GII); etching with 48% hydrofluoric acid for 2 minutes (GIII), and nitric acid/hydrofluoric acid etching for 2 minutes (GIV). The surface roughness (Ra) test was performed, followed by AFM analysis. The results were analyzed by ANOVA and the Tukey test, with the level of significance set at a=.05.

**Results:**

The surface treatment with acid solutions (0.16 ± 0.02-GIII; 0.11 ± 0.01-GIV) promoted a significant increase in roughness, with higher mean Ra values of Y-TZP (μm) compared to control (0.06 ± 0.01-GI) (*p* >.05), and lower values compared to the conventional method (0.21 ± 0.06-GII). The aluminum oxide particle treatment resulted in deep microretentions forming sharp Y-TZP peaks compared to only microretentions with acid solution treatments.

**Conclusions:**

All Y-TZP treatments effectively promoted microretention in the ceramic. Hydrofluoric acid (48%) proved to be more effective in increasing the Ra of Y-TZP than the nitric acid/hydrofluoric acid treatment. Atomic force microscopy images revealed that both acid solutions modified the surface of the Y-TZP in a uniform manner.

** Key words:**Zirconia, surface modification, roughness, yttria-stabilized tetragonal zirconia polycrystal.

## Introduction

The growing demand for esthetics has led dental professionals to make an increasing use of ceramic systems for oral rehabilitations due to their properties, such as translucence, chemical stability, fluorescence, biocompatibility, compressive strength, and a coefficient of thermal expansion closely similar to that of natural teeth (1-3). However, one of the main causes of unsuccessful rehabilitations when using these systems is cementation failure. This factor may be mainly related to insufficient treatment of the internal surface of the ceramic before the clinical cementation procedure.

Among the surface treatment options, etching with hydrofluoric acid at different concentrations is the method most frequently used for ceramics that contain a vitreous phase. Internal airborne particle abrasion of the surface with aluminum oxide particles of different sizes and the application of amphoteric agents (silane) may also be used (1,4).

For zirconia oxide-based ceramics, airborne aluminum particle abrasion plays an important role in improving bond strength by increasing surface roughness and removing contaminating substances (4,5) when used in combination with a silane agent which will permeate the irregularities of the ceramic surface, facilitating the bonding process (6). However, there still is no consensus about the best surface treatment method to use before the adhesive cementation of dental prostheses made of a zirconia oxide-based ceramic material since the microporosities induced by airborne particle abrasion may act as crack-initiators and weaken the ceramic restoration (7,8). Moreover, the airborne particle abrasion systems Rocatec, Silicoater MD and Er-YAG laser require special equipment, increasing operating costs (9).

Therefore, it is necessary to investigate new methodologies that may improve the long-term results and that do not interfere with the properties of ceramics. The purpose of the present study was to evaluate the effect of a conventional method and of surface treatment with acid solutions on the surface roughness of an yttrium-tetragonal zirconia polycrystal (Y-TZP)-based ceramic. The null hypothesis was that the different surface treatments would not affect the surface roughness of the tested zirconia.

## Material and Methods

-Sample

Forty block-shaped specimens (5.0 x 5.0 mm and 2.0 mm in thickness) of a ceramic reinforced with zirconia oxide and stabilized with Y-TZP (Lava, 3M ESPE St. Paul, MN, USA) were fabricated using a metal matrix. The specimens were divided into 4 groups according to the surface treatment method: no treatment-control (GI), airborne 110 µm aluminum oxide particle abrasion for 1 minute-conventional method (GII), etching with 48% hydrofluoric acid for 2 minutes (GIII), and nitric acid/hydrofluoric acid etching for 2 minutes (GIV). The one factor evaluated was surface treatment for the experimental groups (n=10).

The ceramic blocks were individually fixed in a silicone matrix and filled with colorless self-polymerizing acrylic resin (Vipi Flash, Vipi, São Paulo, Brazil). The blocks were polished with silicone oxide abrasive paper grains 600 and 1200 in a vertical polishing machine (Ecomet 300PRO; Buehler, Lake Bluff, IL, EUA) and felt disc with diamond paste. The acid solutions were applied with the aid of a pipette for the time determined and all specimens were then washed with distilled water.

After polishing and surface treatment, the specimens were submitted to surface roughness measurement using the Dektak III profilometer (Veeco, New York, USA). The appliance was calibrated with a measurement filter at 0.25 mm (cut-off), readout speed of 0.1 mm/sec and evaluation length of 1.25 mm. Three consecutive measurements were made in different regions of the specimens (central, right, and left) and the arithmetic mean roughness (Ra) expressed as µm units was obtained.

After the roughness measurement, one specimen of each group was characterized by atomic force microscopy (AFM, Dimension Icon, Bruker, Billerica, MA, USA). For surface characterization, each specimen was metallized before the reading procedure. The test was performed in an area of 10 µm2, number of lines 512, with a 300 nm data scale. The readout speed ranged from 0.39 to 1.0 Hz according to the characteristics of each test specimen. The images obtained were manipulated for transformation from 2D into 3D. Nanoscope 5.12r5 software (Nanoscope III - Digital Instruments, USA) was used, with care taken to maintain the same angle of light incidence and rotation.

-Data analysis

The quantitative surface energy data were analyzed by 1-way repeated-measures analysis of variance (ANOVA), followed by the Tukey HSD test (a=.05). AFM images were compared visually among groups.

## Results

**Figure 1:**
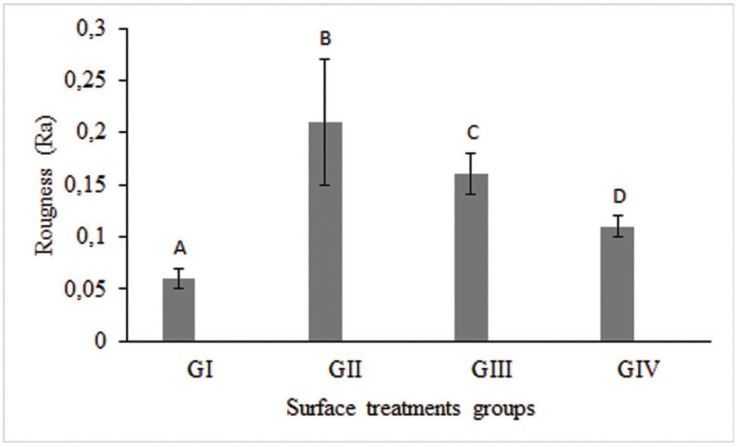
Comparison of Ra among groups tested with different surface treatments. Different uppercase letters indicate statistical differences (*p*<.05) between groups.

The mean roughness values obtained were analyzed statistically and ranged from 0.06 µm to 0.21 µm (Fig. 1). The airborne aluminum particle abrasion (0.21 ± 0.06-GII) promoted a significant increase in mean roughness values (Ra, μm) compared to control (0.06 ± 0.01-GI). However, the surface treatment with acid solutions (0.16 ± 0.02-GIII; 0.11 ± 0.01-GIV) also showed significantly higher mean roughness values (*p*<.05) compared to control, but significantly lower values (*p*<.05) compared to the conventional method-GII. Furthermore, treatment with 48% hydrofluoric acid (0.16 ± 0.02-GIII) induced significantly higher roughness values (*p*<.05) than treatment with nitric acid/hydrofluoric acid (0.11 ± 0.01-GIV) (Fig. 1).

**Figure 2:**
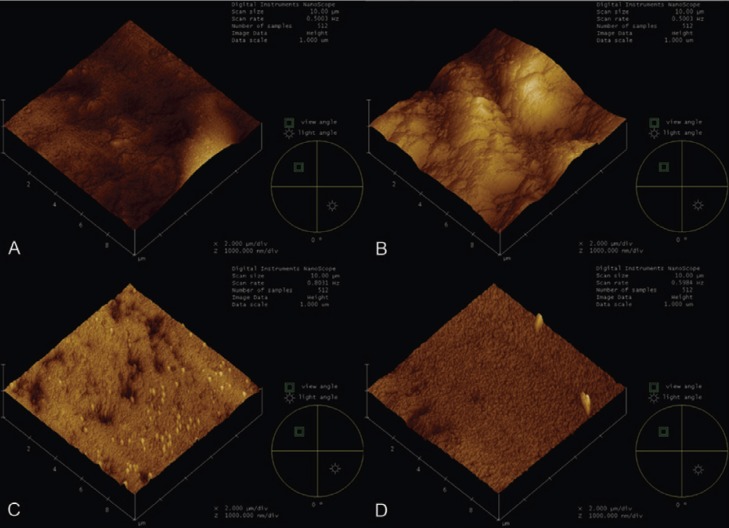
A. GI- Control: without any previous treatment. B. GII- Conventional method: Airborne Al2O3 particle abrasion for 1 minute. C. GIII- Acid tretament: 48% hydrofluoric acid for 2 minutes. D. GIV- Acid tretament: nitric acid/hydrofluoric acid for 2 minutes.

Topographic modifications on the zirconia surface were identified through AFM images. GII specimens showed images with deep microretentions forming sharp peaks with the presence of irregularities similar to surface deteriorations and different from the control group (GI, Figs. 2A and B). GIII and GIV specimens showed images with uniformly distributed microretentions (Figs. 2C and D).

## Discussion

The null hypothesis was rejected because the all-surface treatment methods changed the surface topography of the tested ceramics. Zirconia is characterized as a ceramic with three crystallographic configurations, namely: monoclinic (M), tetragonal (T) and cubic (C). The addition of stabilizing oxides has been recommended in order to stabilize the zirconia phases. The most frequently used are Yttrium (Y2O3) and Cerium (CeO2). In order to be used in Dentistry, this ceramic must be shown to have crystals in the tetragonal phase under ambient temperature and pressure conditions (4,10,11).

The bond of ceramic restorations to dental substrates allows the dissipation of stress generated by masticatory function, so that the tooth and restoration function as one integrated system (12). In view of this, it is important for the dentist to perform previous treatment of the internal surface of ceramics, according to the approach used in the present study. The surface treatments with hydrofluoric acid and airborne aluminum oxide particle abrasion (Al2O3) have been shown to be efficient for feldspathic ceramics and those reinforced with lithium disilicate (4,8,13). For aluminum oxide and zirconia-based ceramics, these methods were unable to modify the morphological characteristics of their surfaces (2,12).

Some studies have found that the roughness of zirconia-based ceramic was increased when silicatization was used (airborne particle abrasion with coating of Al2O3 particles with a silica layer) or even airborne Al2O3 particle abrasion only (2,10,14). Is it known that the powder particle size used in airborne particle abrasion is important as regards surface treatment. The impact speed of particles on the substrate causes the smaller sized particles (30 or 50 μm) to have a more aggressive action, producing gaps or deeper surface defects. The larger particles (100 or 110 μm) may not have a significant effect on increasing the depth of surface microretentions in ceramic because of its high resistance (15-18).

In our study we observed that the surface of the tested ceramic was modified by airborne Al2O3 particle abrasion and acid solutions, with higher mean roughness compared to control (Fig. 1). However, the acid solution surface treatments showed AFM images with uniformly distributed microretentions (Fig. 2C,D). Some authors have reported that airborne particle abrasion of zirconia oxide-reinforced ceramic systems may be effective only at first and may not be stable, with a significant reduction in bond strength after different storage periods and thermal cycling (8,11). The mechanical stress caused by airborne particle abrasion induces transformations from the tetragonal to the monoclinic phase, later resulting in compressive stress (11).

The rate of adhesive failure in ceramics is of the order of 2.3% to 8%, so that the integrity of the cementing agent on the ceramic surface becomes important in order to guarantee the longevity of restorations. In view of this, the failures observed on the cement surface indicate the need for efficient etching methods in order to increase the bond strength at the tooth-restoration interface (5,6,12,19). Surface treatment allows the physical and/or chemical bond to the ceramic substrate to be effected (1,20). If not treated, this ceramic may behave as an inert substrate, with low surface energy and wettability.

Pretreatment of zirconia oxide-based ceramic must be performed but must not compromise the long-term restoration longevity. Creating microretentions in a highly resistant ceramic substrate with a view to preparing it for cementation is not an easy task, because its surface is compact, hard and difficult to change (9,21). Additional studies should be performed to determine the efficacy of the surface treatment methods for zirconia oxide-based ceramics, particularly with the use of stronger acids.

## Conclusions

On the basis of the present results, we may conclude that: (1) all the surface treatments performed were effective in promoting microretentions in the Y-TZP as determined by AFM imaging. (2) The Y-TZP treated with aluminum oxide particle abrasion (conventional method) showed a significantly higher Ra and deep microretentions on the topographic surface. (3) On the other hand, the acid solution treatments statistically affected the Ra compared to control and both modified the topography of the Y-TZP in a uniform manner. (4) Treatment with 48% hydrofluoric acid proved to be more effective in increasing the Ra of the Y-TZP compared to the nitric acid/hydrofluoric acid treatment.
